# Technological advancements and the use of key performance indicators in hospital management: a critical-conceptual review

**DOI:** 10.3389/frhs.2026.1780458

**Published:** 2026-03-18

**Authors:** Aydin Teymourifar

**Affiliations:** Department of Industrial Engineering, Istanbul Sabahattin Zaim University, Istanbul, Türkiye

**Keywords:** digital health technologies, hospital management, key performance indicators (KPIs), KPI limitations, performance measurement

## Abstract

Despite extensive critiques of key performance indicator (KPI)-based performance measurement and rapid advances in digital health technologies, limited research has examined how such technological and data capabilities intersect with the fundamental limitations of hospital KPIs. This study addresses this gap through a critical-conceptual review that synthesizes the literature on hospital performance measurement and healthcare technology. Focusing on the main critiques of KPIs across six major categories of hospital performance, financial performance, timeliness of care and access, patient experience, quality of care and patient safety, workforce-related performance, and resource utilization and operational efficiency, the review analyzes how technological advancements influence the use of commonly applied hospital and emergency department KPIs. The paper discusses that technological and data-related advancements consistently mitigate technical constraints on KPI use by improving data completeness, timeliness, and system integration. However, these improvements do not resolve the core limitations of KPI-based performance frameworks. Across the main critiques of KPIs, enhanced technologies are shown not to resolve all limitations and may even intensify problems related to metric dominance, inequitable benchmarking, and performance paradoxes. Overall, the review demonstrates that technology, as a standalone intervention, does not improve hospital performance. By conceptually linking established KPI critiques with technological advancements across performance domains, this study closes an important gap in the literature. The review furthermore provides a structured foundation for future research and managerial decision-making on value-driven hospital performance measurement.

## 1 Introduction

Performance measurement is a cornerstone of contemporary healthcare governance, as hospitals worldwide face growing demands to demonstrate accountability, operational efficiency, and high-quality care (1, 2). Key Performance Indicators (KPIs) play a critical role in systematically monitoring performance across multiple domains, including financial performance, timeliness of care, patient experience, quality of care, workforce-related outcomes, and resource utilization (3, 4). Consequently, policymakers and accrediting bodies widely endorse KPIs as essential instruments for evidence-based decision-making and strategic performance improvement (5–10).

Nevertheless, the use of KPIs in healthcare remains contested. While they are intended to support accountability and improvement, critics argue against them (11). The concerns are not merely theoretical; prominent examples illustrate how such limitations manifest in practice. For example, the Standardized Mortality Ratio (SMR) has been criticized for producing misleading inter-hospital comparisons due to variations in case mix and data coding (12–14). Similarly, the U.S. Hospital Readmissions Reduction Program (HRRP), though successful in reducing readmissions, has been associated with increased mortality among heart failure patients (15–18). Pay-for-Performance (P4P) has been criticized for generating unintended behavioral distortions (19). England's Quality and Outcomes Framework (QOF) provides another example: while it improved selected metrics, it also heightened administrative burden without producing proportional gains in genuine care quality (20–22).

Table 1 synthesizes the main critiques of KPI-based hospital performance measurement identified in the literature, grouping them into recurring thematic limitations and their key implications.

**Table 1:** Some of the common critiques of the use of KPIs in hospital management.

Theme/limitation	Core Issue	Key implications	Representative references
Measurability Over Meaning (Indicator Selection Bias)	Performance measurement often privileges what is easily quantifiable over what is genuinely meaningful, leading to conceptually narrow KPI sets.	Some key quality domains (e.g., patient experience, care coordination, contextual determinants) are under-measured, producing a distorted picture of hospital effectiveness.	Eddy (23); Likierman (24); Mannion & Braithwaite (11); Plebani (25)
Unintended Behavioral Consequences of KPI Regimes	KPI-driven systems, especially when linked to incentives (e.g., P4P), can distort behavior as actors optimize measured targets rather than underlying outcomes.	Gaming, tunnel vision, and effort substitution; artificial metric improvements; potential neglect of complex/high-risk patients; reduced managerial and clinical efficiency.	Bevan & Hood (19); Petersen et al. (26); Van Herck et al. (27); Franco-Santos & Otley (28); Bardach & Cabana (29); Li & Evans (30)
Inequitable and Methodologically Fragile Benchmarking	Standardized KPIs used for cross-hospital comparisons (e.g., SMR) may not adequately adjust for case mix, socioeconomic context, institutional differences, and coding practices.	Misleading inter-hospital comparisons; unfair penalization of providers serving vulnerable populations; weakened interpretive validity of benchmarking results.	Homa-Lowry & Wooster (31); Glance et al. (13); van Gestel et al. (14); Roessler et al. (32); Bardach & Cabana (29); Li & Evans (30)
Context and Complexity Blindness	Conventional KPI frameworks often rely on linear, reductionist representations (including traditional quality assessment models), under-capturing system dynamics and interdependencies.	Incomplete understanding of system-wide performance and efficiency; suboptimal managerial and policy decisions in complex, adaptive healthcare settings.	Braithwaite et al. (33); Donabedian (6); Araz (34); Woolf (35); Dyer et al. (36); Bardach & Cabana (29, 30)
Indicator Decay (Performance Paradox)	Over time, indicators can lose validity as organizations adapt their behavior to meet targets rather than improve actual performance (the indicator decay/performance paradox).	Short-term compliance and apparent gains that mask limited real improvement; declining indicator informativeness; potential long-run efficiency losses.	Neely & Bourne (37); Bourne et al. (38); Chrystal et al. (39); Lewis (40); Garrubba et al. (41); El-Mhamdi & Hoang (42); Franco-Santos & Otley (28)
Design Responses and Mitigation Strategies	To address the above limitations, measurement systems should be adaptive, multidimensional, and context-sensitive rather than narrowly target-driven.	Composite frameworks integrating quantitative and qualitative metrics; expert panels for KPI design; broader KPI portfolios across diverse patient groups; balanced process vs outcome measures; risk-adjusted and stratified benchmarking; regular KPI review, as well as renewal systems-based analytics like systems thinking, simulation.	Kelley & Hurst (3); Eijkenaar (43); Eijkenaar et al. (44); Roessler et al. (32); Araz (34)

On the other hand, recent advancements in digital health technologies, including the Internet of Medical Things (IoMT), artificial intelligence (AI), radio-frequency identification (RFID), real-time location systems (RTLS), and intelligent dashboards, can present new opportunities for transforming KPI monitoring and interpretation (45–47). These tools can enhance the performance insights, enabling more responsive, patient-centered decision-making (48, 49). Nevertheless, scholars emphasize that technological augmentation can be a double-edged sword (50) and must be balanced with human oversight to ensure that performance systems remain transparent, ethical, and contextually grounded (51).

In this context, we pose two primary research questions, both grounded in prior works in the literature:

1. Are technical and data-related challenges a key reason why hospitals struggle to use KPIs efficiently (9, 41)?
2. Can technological advancements help hospitals reduce these challenges and use KPIs more effectively (52, 53)?

Table 2 maps the core critiques of KPI-based hospital performance measurement to specific data and measurement limitations, highlighting the potential and limitations of technological advancements in addressing these critiques.

**Table 2:** Mapping core critiques of KPI-based hospital performance measurement to technological and data-related advancements.

Core critique	Data/measurement limitation	Potential role of technology (and limits)
Indicator selection bias	Limited data availability and weak measurement infrastructure encourage reliance on easily quantifiable proxies rather than clinically meaningful indicators (11, 23–25, 41).	Enhanced data capture (e.g., patient-reported outcomes, pathway-level data) and analytics can support a richer set of KPIs, though conceptual judgment remains essential (9).
Unintended behavioral consequences	Narrow datasets and simplistic indicators increase susceptibility to gaming and target optimization (19, 26–30).	Broader KPI portfolios and the balancing of metrics, enabled by improved data systems, may reduce manipulation, but incentive design and governance remain decisive (11).
Inequitable comparisons	Poor coding quality, missing risk factors, and limited contextual variables undermine the validity of risk adjustment and benchmarking (13, 14, 31, 32).	Interoperability, standardized data, and advanced risk-adjustment models can improve fairness, but structural context cannot be fully neutralized (9, 41).
Context and complexity blindness	Siloed systems and low-granularity data promote linear, reductionist performance views (6, 33–35).	Integrated platforms, systems analytics, simulation, and process mining better capture interdependencies and contextual dynamics (41).
Indicator decay (performance paradox)	Static KPIs and slow feedback loops reduce the relevance of indicators over time (28, 37–41).	Adaptive analytics, drift detection, and routine KPI refresh cycles can help sustain indicator validity.
Mitigation strategies (cross-cutting)	Data and technical limitations constrain multidimensional, context-sensitive performance frameworks (3, 32, 43).	Technology enables adaptive systems, but effectiveness depends on governance, transparency, and incentive alignment (11, 41).

### 1.1 The literature gap and the study's contribution

Despite extensive scholarship on hospital performance measurement and parallel advances in digital health technologies, their intersection remains insufficiently examined. Foundational critiques of KPI-based regimes, including gaming, inequitable benchmarking, metric dominance, and indicator decay, are well established (11, 19, 28). Importantly, recent empirical work continues to document these dynamics in contemporary and digitally enabled performance systems (30, 32, 42), suggesting that such limitations are structural rather than merely historical.

At the same time, digital transformation is frequently portrayed as a solution to performance measurement challenges through enhanced data quality, interoperability, predictive analytics, and real-time dashboards (45, 46). However, little research has systematically evaluated whether these technological advancements address the structural limitations of KPI regimes or primarily refine their technical precision.

This review contributes by:

1. **Systematically integrating two largely parallel research streams**, critiques of KPI-based hospital performance measurement and advances in digital health technologies.
2. **Developing a cross-domain analytical framework** that maps six core KPI critiques across financial, timeliness, patient experience, quality and safety, workforce, and resource utilization domains.
3. **Distinguishing between technical measurement constraints and structural governance limitations**, clarifying which limitations technology can mitigate (e.g., data completeness, interoperability) and which remain embedded in incentive design, benchmarking structures, and performance regimes.
4. **Demonstrating that digital technologies may amplify, rather than resolve, longstanding KPI distortions**, particularly in relation to metric dominance, inequitable comparisons, and indicator decay.

Rather than assuming that improved instrumentation leads to improved performance management, this review repositions digital health technologies as amplifiers of existing KPI regimes, whose ultimate effects depend on governance design, incentive alignment, and contextual interpretation.

The remainder of the article is structured as follows. Section 2 presents the methodological approach. Section 3 provides the analytical synthesis across KPI domains and incorporates a discussion of the findings and their implications. Section 4 offers concluding remarks and directions for future research.

## 2 Materials and methods

This study adopts a critical-conceptual review approach, emphasizing analytical synthesis and conceptual integration rather than exhaustive aggregation of evidence. In line with established review typologies, critical reviews differ from systematic reviews and narrative overviews by prioritizing interpretive depth and conceptual development (54–56). Such approaches are particularly suitable for examining emerging or cross-cutting topics that require theoretical integration (57, 58). This review follows a similar application of the critical-conceptual strategy within healthcare management research (50).

### 2.1 Two-Phase literature exploration process

The conceptual framework was developed through a structured two-phase literature exploration process designed to ensure both theoretical grounding and contemporary relevance.

#### 2.1.1 Phase 1: exploratory identification of core KPI critiques

In the first (exploratory) phase, a broad search was conducted to identify recurring critiques of KPI-based performance measurement in hospital settings. Searches were performed in major academic databases (Scopus, Web of Science, PubMed) using combinations of keywords such as hospital performance measurement, KPI limitations, performance paradox, gaming, risk adjustment, and healthcare complexity. No strict temporal restriction was imposed, as both foundational and contemporary contributions were relevant for identifying structural critiques.

Through iterative screening, close reading, and thematic clustering, recurring categories of critique were identified. These included:

1. **Measurability bias and indicator selection distortions**, where performance systems privilege quantifiable metrics over meaningful constructs (6, 11, 23–25);
2. **Unintended behavioral consequences and gaming dynamics**, particularly under incentive-linked regimes (19, 26, 28, 29);
3. **Inequitable and methodologically fragile benchmarking**, especially in relation to case-mix adjustment and coding variation (13, 14, 31, 32);
4. **Context and complexity blindness**, reflecting reductionist performance representations in complex adaptive healthcare systems (33–35);
5. **Indicator decay and performance paradox dynamics**, whereby adaptation to metrics erodes their informational value over time (28, 37, 38, 41).

These critiques formed the conceptual foundation of the analytical framework used in the subsequent phase.

#### 2.1.2 Phase 2: focused review of technological and data-related advancements

In the second (focused) phase, the search targeted technological and data-related advancements relevant to KPI measurement, monitoring, and interpretation in hospital contexts. Keywords included digital health, AI in healthcare, RFID, IoT, EHR integration, real-time dashboards, and healthcare analytics, often combined with performance-related terms.

Greater emphasis was placed on recent contributions reflecting the rapid evolution of digital infrastructures and AI-enabled analytics (45, 46, 52, 53). At the same time, policy and governance sources relevant to KPI data systems and measurement infrastructures were retained (9, 41).

Selected grey literature was included where directly relevant to interoperability standards, national KPI frameworks, or governance mechanisms shaping performance measurement systems.

#### 2.1.3 Analytical integration across phases

Across performance domains, each KPI was examined across three dimensions as follows:

1. the extent to which technological advancements enhance technical robustness and data quality;
2. how these advancements interact with established KPI critiques (e.g., gaming, inequitable benchmarking, indicator decay);
3. and the governance implications for adaptive and context-sensitive performance management.

This approach enabled systematic cross-domain comparison while preserving the interpretive depth characteristic of critical-conceptual reviews (54, 57, 58).

### 2.2 Advantages and limitations of the critical-conceptual approach

This study adopts a critical-conceptual review methodology, which is particularly suitable for examining cross-cutting and theoretically layered questions (55, 57, 58). Unlike systematic or purely bibliometric reviews that prioritize exhaustive aggregation and quantitative mapping of literature, a critical-conceptual review emphasizes interpretive synthesis and conceptual integration (50).

This approach offers several advantages in the context of the present study:

1. **Conceptual integration across parallel research streams:** The literature on KPI-based performance measurement and the literature on digital health technologies have largely evolved independently. A critical-conceptual review enables structured integration of these streams rather than an isolated summary.
2. **Evaluation of structural vs. technical limitations:** The central research question concerns whether technological advancements address structural governance critiques of KPI regimes. Such normative and interpretive distinctions require analytical synthesis beyond descriptive aggregation.
3. **Cross-domain comparative analysis:** The study maps six recurring critiques across multiple performance domains (financial, clinical, experiential, workforce, and operational). This comparative and framework-building objective aligns with the strengths of conceptual review methodologies.
4. **Suitability for emerging digital transformation debates:** Given the rapidly evolving nature of healthcare analytics and AI-enabled infrastructures, rigid systematic inclusion criteria may prematurely constrain analysis. A critical-conceptual approach allows evaluation of both established theoretical critiques and contemporary technological developments within a unified framework.

At the same time, this methodology involves interpretive judgment in selecting literature and synthesizing themes. While efforts were made to ensure transparency and conceptual coherence, alternative mappings or interpretations may emerge as empirical validation accumulates and digital infrastructures continue to evolve.

## 3 Results and discussion

To examine whether, and in what ways, technological advancements enhance the use of KPIs in hospital management, this review is structured around six core domains of hospital performance measurement: financial performance; timeliness of care and access; patient experience; quality of care and patient safety; workforce-related performance; and resource utilization and operational efficiency. These domains align with widely adopted hospital KPI frameworks and collectively capture the inherently multidimensional nature of hospital performance.

Within each domain, commonly used hospital-level KPIs and their emergency department (ED) equivalents, where applicable, are examined using a consistent analytical lens. Specifically, the review assesses how recent technological and data-related advancements influence the technical robustness and interpretability of each KPI, and how these KPIs interact with well-documented limitations of KPI-based performance measurement, including incentive effects, contextual sensitivity, and indicator decay.

### 3.1 Financial performance KPIs

Within the financial performance domain, revenue-related indicators are typically used to assess alignment between clinical activity and reimbursement mechanisms. The first indicator considered is revenue per admission.

#### 3.1.1 Revenue per admission

Revenue per admission, operationalized in emergency care as revenue per ED visit, reflects the average financial return generated per patient encounter and is influenced by case mix, reimbursement mechanisms, and service intensity (59, 60). The indicator is commonly used to assess alignment between clinical activity and revenue generation and to identify variations across care settings.

Table 3 summarizes how recent technological advancements influence the measurement and interpretation of revenue per admission, and how they interact with core critiques of KPI-based performance measurement.

**Table 3:** Revenue per admission: technological enablers and implications for core critiques of KPI-based hospital performance measurement.

Dimension	Content
KPI focus	Average revenue per patient encounter; a proxy indicator of alignment between activity and reimbursement (59, 60).
Technological advancements discussed	RFID- and IoT-enabled billing automation for automated charge capture (61); integrated Electronic Health Record (EHR)–billing systems enabling real-time documentation-to-charge linkage (62).
Potential benefits	**Technical and data-related challenges:** improved data completeness, timestamping, and linkage between clinical events and financial records, reducing missing or inconsistent charge data.
	**Measurability over meaning:** reduced measurement error by capturing delivered-but-unrecorded services, improving interpretability.
Key risks and limitations	**Unintended behavioral consequences:** intensified incentives to optimize billable activity rather than value (documentation inflation, over-coding).
	**Indicator decay:** workflow adaptation to maximize revenue metrics reduces validity over time.
	**Context/inequitable comparisons:** payer mix and reimbursement structures remain unaddressed, potentially legitimizing methodologically fragile comparisons.

#### 3.1.2 Revenue-to-cost ratio

The revenue-to-cost ratio provides an aggregate measure of financial efficiency by comparing income generated from clinical services with the resources consumed to deliver them. It is widely used to evaluate service-line sustainability, the effectiveness of cost control, and the financial impact of operational redesign or resource reallocation (63).

Table 4 outlines the technological enablers associated with the revenue-to-cost ratio.

**Table 4:** Revenue-to-cost ratio: technological enablers and implications for core critiques of KPI use.

Dimension	Content
KPI focus	Aggregate financial efficiency indicator linking revenues to resource consumption (63).
Technological advancements discussed	RFID-based asset tracking and utilization monitoring (64–66); IoT-enabled energy monitoring and infrastructure optimization (67); integrated EHR–financial systems for cost attribution (62).
Potential benefits	**Technical and data-related challenges:** improved visibility into cost drivers via enhanced granularity, accuracy, and timeliness of cost data (9, 41).
	**Context and complexity blindness:** more precise representation of how costs accumulate across workflows, assets, and infrastructure (33).
Key risks and limitations	**Measurability over meaning:** improved precision does not address neglect of patient outcomes, equity, or experience.
	**Unintended behavioral consequences:** incentives for aggressive cost-cutting that may compromise clinical appropriateness.
	**Inequitable benchmarking:** continued sensitivity to reimbursement structures and case mix.
	**Indicator decay:** apparent efficiency gains may mask longer-term risks to quality and system resilience.

#### 3.1.3 Cost per admission

Cost per admission and its ED equivalent, the cost per ED visit, measure the average expenditure incurred per patient episode. These indicators are central to evaluating operational efficiency, comparing hospital performance, and identifying opportunities to reduce unnecessary utilization without compromising quality (68–70).

Table 5 examines how technological and data-related advancements affect the use of cost per admission.

**Table 5:** Cost per admission: technological enablers and implications for core critiques of KPI use.

Dimension	Content
KPI focus	Average cost per patient episode; proxy for operational efficiency and cost containment (68–70).
Technological advancements discussed	RFID-based asset tracking (64–66); IoT-enabled energy monitoring (67); telehealth for low-acuity care substitution (7–71]); integrated EHR–financial systems for episode-level cost attribution (62).
Potential benefits	**Technical and data-related challenges:** reduced fragmentation and improved episode-level cost attribution (9, 41).
	**Context and complexity blindness (partial):** more granular insight into cost accumulation along care pathways.
Key risks and limitations	**Measurability over meaning:** cost reductions may not reflect value creation.
	**Unintended behavioral consequences:** incentives for premature discharge, underuse of diagnostics, or avoidance of complex patients.
	**Inequitable benchmarking:** persistent sensitivity to case mix and socioeconomic context.
	**Indicator decay:** workflow adaptation may erode the measure's validity as a proxy for sustainable performance.

#### 3.1.4 Claims denial rate

The claims denial rate captures the proportion of submitted reimbursement claims rejected by payers due to documentation gaps, coding errors, or administrative inconsistencies. High denial rates are associated with revenue leakage, delayed cash flow, and increased administrative burden in both inpatient and emergency care settings (62, 74).

Table 6 reviews the role of emerging technologies in measuring claims denial rates.

**Table 6:** Claims denial rate: technological enablers and implications for core critiques of KPI use.

Dimension	Content
KPI focus	Share of rejected reimbursement claims; indicator of billing accuracy and documentation quality (62, 74).
Technological advancements discussed	AI-assisted clinical documentation and coding support (74–76); integrated EHR–billing systems (62); RFID-enabled automatic capture of resource utilization (77–79).
Potential benefits	**Technical and data-related challenges:** improved documentation accuracy, completeness, and traceability, strengthening KPI reliability (9, 41).
	**Measurability over meaning (limited):** reduced administrative noise so the KPI reflects genuine billing issues.
Key risks and limitations	**Unintended behavioral consequences:** documentation inflation and defensive charting.
	**Context and complexity blindness:** payer-specific rules limit comparability across hospitals and EDs.
	**Indicator decay:** compliance-driven optimization may erode substantive improvements.
	**Equity concerns:** higher denial rates may persist for hospitals serving complex or disadvantaged populations.

#### 3.1.5 Operational margin

Operational margin reflects the difference between operating revenues and operating expenses and is widely used as a high-level indicator of financial sustainability. In hospital settings, it is sensitive to cost structures, patient mix, reimbursement policies, and efficiency initiatives, and is frequently monitored to support strategic planning (9).

Table 7 summarizes how technological integration influences the operational margin indicator.

**Table 7:** Operational margin: technological enablers and implications for core critiques of KPI use.

Dimension	Content
KPI focus	High-level proxy for financial sustainability; difference between operating revenues and expenses (9).
Technological advancements discussed	Integrated EHR–financial and management dashboards (9, 80, 81); RFID-based asset utilization optimization (64–66); IoT-enabled infrastructure and energy management (67); telehealth and alternative care delivery models (71–73).
Potential benefits	**Technical and data-related challenges:** improved data timeliness, integration, and interpretability (9, 41).
	**Context and complexity blindness (partial):** more explicit linkage between margin performance and underlying cost drivers.
Key risks and limitations	**Measurability over meaning:** excessive focus on financial sustainability may overshadow patient-centered outcomes, equity, and resilience.
	**Unintended behavioral consequences:** cost-cutting affecting staffing, service availability, or quality.
	**Inequitable benchmarking:** sensitivity to patient mix and reimbursement context.
	**Indicator decay:** short-term margin gains may undermine long-term sustainability and distort informational value.

Across financial KPIs, technological and data-related advancements consistently mitigate technical and measurement limitations. Yet, they do not resolve, and may amplify, critiques linked to incentive design, governance, and contextual heterogeneity. These findings reinforce the need to embed technological innovation within adaptive governance frameworks to prevent gaming, inequitable benchmarking, and indicator decay.

### 3.2 Timeliness of care/access KPIs

Timeliness of care and access KPIs are used to measure speed, responsiveness, and delays across patient pathways, supporting early risk identification, flow efficiency, congestion and bottleneck detection, and improved clinical outcomes in time-sensitive conditions such as stroke, sepsis, and acute cardiac events (9, 82–85). Delays in assessment and treatment are consistently associated with adverse patient outcomes and operational inefficiencies.

Several timeliness indicators are widely applied at the hospital level, with closely related equivalents used in ED settings. Each KPI is examined below through a common analytical lens that links technological and data-related advancements to core critiques of KPI-based performance measurement.

#### 3.2.1 Time to initial clinical assessment

Time to initial clinical assessment measures the interval between patient arrival and the first structured evaluation by a clinical team. In EDs, the equivalent indicator is time to triage, which captures the time from arrival to initial acuity assessment and plays a critical role in early risk stratification and prioritization. Delays at this stage are associated with downstream congestion, prolonged waiting times, and compromised patient safety, particularly in high-acuity environments (82, 83).

Table 8 summarizes the technological enablers relevant to time to initial clinical assessment and evaluates their implications for addressing measurement challenges.

**Table 8:** Time to initial clinical assessment: technological enablers and implications for core critiques of KPI use.

Dimension	Content
KPI focus	Interval from arrival to first clinical or triage assessment; proxy for early access and risk stratification (82, 83).
Technological advancements discussed	RFID- and RTLS-based patient tracking systems (66, 86, 87); IoT-enabled self-check-in kiosks and digital registration systems (88, 89); AI-enabled real-time flow dashboards (81, 90, 91).
Potential benefits	**Technical and data-related challenges:** automated timestamping and real-time integration improve accuracy, consistency, and timeliness of measurements (9, 41).
	**Context and complexity blindness (partial):** real-time visibility enables interpretation of delays relative to demand surges, staffing, and spatial constraints.
Key risks and limitations	**Measurability over meaning:** focus on threshold compliance may not ensure clinically meaningful assessment.
	**Unintended behavioral consequences:** rushed or superficial assessments to “stop the clock.”.
	**Indicator decay:** adaptation of workflow definitions erodes validity.
	**Equity and contextual bias:** sensitivity to acuity mix and arrival patterns limits comparability across hospitals and EDs.

#### 3.2.2 Time to first provider assessment (door-to-provider time)

Time to first provider assessment reflects how quickly a qualified clinician evaluates patients after arrival. In EDs, door-to-provider time captures the efficiency of registration, triage, and early workflow coordination. Shorter times are associated with improved patient experience, reduced rates of leaving without being seen, and earlier initiation of diagnostic pathways (9, 92).

Table 9 outlines how data integration and real-time analytics affect the measurement of time to first provider assessment.

**Table 9:** Time to first provider assessment: technological enablers and implications for core critiques of KPI use.

Dimension	Content
KPI focus	Interval from arrival to first clinician contact; proxy for access to care and early engagement (9, 92).
Technological advancements discussed	RTLS and RFID-based staff and patient tracking (66, 87); AI-enabled operational dashboards and predictive analytics (81, 90, 91); integrated EHR workflow orchestration systems (9).
Potential benefits	**Technical and data-related challenges:** improved consistency and real-time measurement of arrival, assignment, and first-contact events (9, 41).
	**Context and complexity blindness (partial):** staffing levels, acuity, and spatial constraints incorporated into interpretation.
Key risks and limitations	**Measurability over meaning:** reduced emphasis on the quality or appropriateness of the encounter.
	**Unintended behavioral consequences:** superficial “provider touches” to meet thresholds. **Indicator decay:** redefinition of contact points undermines validity.
	**Equity and benchmarking limitations:** sensitivity to acuity mix, arrival mode, and surge conditions.

#### 3.2.3 Time to treatment

Time to treatment measures the interval from diagnosis or clinical decision to initiation of definitive care. It is particularly critical in time-sensitive pathways such as sepsis, stroke, and myocardial infarction. Shorter intervals are strongly associated with improved survival and reduced morbidity (84, 85, 93).

Table 10 reviews technological contributions to measuring time to treatment and assesses their potential to improve responsiveness.

**Table 10:** Time to treatment: technological enablers and implications for core critiques of KPI use.

Dimension	Content
KPI focus	Interval from diagnosis/decision to treatment initiation; proxy for clinical responsiveness in time-critical pathways (84, 85, 93).
Technological advancements discussed	RFID and RTLS workflow tracking (66, 86, 87, 94); AI-enabled prioritization dashboards (81, 90, 91); IoT-enabled vital-sign monitoring and early warning systems (95–97); EHR-integrated decision support systems (9, 84).
Potential benefits	**Technical and data-related challenges:** accurate, real-time measurement of treatment intervals (9, 41).
	**Context and complexity blindness (partial):** integration of acuity, staffing, and workflow status into interpretation.
Key risks and limitations	**Measurability over meaning:** prioritization of speed over diagnostic certainty.
	**Unintended behavioral consequences:** premature or overly standardized interventions.
	**Indicator decay:** gaming through redefinition of treatment start points.
	**Equity and benchmarking limitations:** sensitivity to complexity and comorbidities across settings.

#### 3.2.4 Hospital length of stay (LOS)

Hospital LOS measures the total duration of an inpatient admission, while ED LOS captures the time from arrival to discharge or admission. Both serve as proxies for care coordination, throughput efficiency, and resource utilization. Prolonged LOS is associated with adverse events, reduced satisfaction, and operational strain (98, 99).

Table 11 presents the technological enablers associated with hospital and ED LOS.

**Table 11:** LOS: technological enablers and implications for core critiques of KPI use.

Dimension	Content
KPI focus	Total time in inpatient or ED care; proxy for coordination, throughput, and resource utilization (98, 99).
Technological advancements discussed	RFID and RTLS patient-flow tracking (66, 86, 87); AI-enabled flow dashboards (81, 90, 91); IoT-enabled diagnostic turnaround monitoring (100, 101); EHR-based bed management and discharge coordination (9, 99).
Potential benefits	**Technical and data-related challenges:** continuous, accurate measurement of movement and delays (9, 41).
	**Context and complexity blindness (partial):** interpretation relative to diagnostics, beds, staffing, and discharge processes.
Key risks and limitations	**Measurability over meaning:** focus on time reduction over clinical appropriateness.
	**Unintended behavioral consequences:** premature discharge or prioritization of low-complexity patients.
	**Indicator decay:** shifting delays outside measured boundaries.
	**Equity and benchmarking limitations:** sensitivity to social determinants and post-acute care availability.

#### 3.2.5 Throughput rate

Throughput rate reflects how efficiently patients progress through assessment, diagnostics, treatment, and disposition. In both hospital and ED contexts, low throughput signals bottlenecks that contribute to crowding, access delays, and reduced system performance (101).

Table 12 summarizes how emerging technologies support throughput measurement and highlights their implications.

**Table 12:** Throughput rate: technological enablers and implications for core critiques of KPI use.

Dimension	Content
KPI focus	Speed and efficiency of patient progression through care pathways; proxy for system coordination (101).
Technological advancements discussed	RFID/RTLS patient and asset flow tracking (66, 86, 87); AI-enabled congestion prediction dashboards (81, 90, 91); IoT-enabled diagnostic turnaround monitoring (100, 101); EHR-based coordination and bed management systems (9, 99).
Potential benefits	**Technical and data-related challenges:** accurate, continuous flow measurement (9, 41).
	**Context and complexity blindness (partial):** interpretation relative to interacting system components.
Key risks and limitations	**Measurability over meaning:** speed prioritized over safety and experience.
	**Unintended behavioral consequences:** cherry-picking low-complexity cases or premature disposition.
	**Indicator decay:** redefinition of process boundaries.
	**Equity and benchmarking limitations:** sensitivity to case mix, arrival patterns, and social determinants.

Across timeliness and access KPIs, technological advancements consistently strengthen measurement precision and real-time visibility. Nevertheless, they do not resolve, and may amplify, critiques related to threshold-driven behavior, tunnel vision, and contextual heterogeneity. Without adaptive governance and safeguards, improved instrumentation risks accelerating indicator decay and inequitable benchmarking rather than improving genuine access and responsiveness.

### 3.3 Patient experience KPIs

Patient experience KPIs capture patient-centered perceptions of care quality and support evaluation of patient-centered care, trust and transparency, service responsiveness, and loyalty and reputational outcomes at both hospital and departmental levels (9, 102, 103). These indicators complement clinical and operational KPIs by reflecting how patients perceive communication, responsiveness, and overall service quality across the care continuum.

Several patient experience KPIs are widely applied at the hospital level, with closely related equivalents used in ED settings. Each KPI is examined below using a common analytical lens that links technological and data-related advancements to core critiques of KPI-based performance measurement.

#### 3.3.1 Patient satisfaction score

The patient satisfaction score is a core hospital-level indicator that captures patients' overall perceptions of care quality, communication, comfort, and trust during inpatient or outpatient encounters. In EDs, the patient satisfaction score reflects experiences of waiting times, communication clarity, perceived competence, and environmental conditions in high-acuity and high-uncertainty settings. Patient satisfaction has been consistently linked to adherence, service utilization, and long-term engagement with healthcare providers (102).

Table 13 examines how digital health technologies expand the measurement of patient satisfaction.

**Table 13:** Patient satisfaction score: technological enablers and implications for core critiques of KPI use.

Dimension	Content
KPI focus	Overall perceptions of care quality, comfort, communication, and trust; ED variant emphasizes wait times, communication clarity, perceived competence, and environmental conditions (102).
Technological advancements discussed	IoT-enabled environmental monitoring (noise, temperature, air quality, crowding) (104–107); RFID/RTLS patient journey tracking and wait-time transparency (108–111); digital feedback kiosks and mobile survey tools (112–114); sentiment analysis and text analytics of patient feedback (115–117).
Potential benefits	**Measurability over meaning:** environmental sensors, journey tracking, and sentiment analysis broaden experience measurement beyond numeric survey scores (118).
	**Technical and data-related challenges:** real-time digital feedback improves completeness, timeliness, and response rates and reduces recall bias (9, 41).
	**Context and complexity blindness:** linking satisfaction to flow and environmental conditions enables contextual interpretation (e.g., crowding, wait times).
Key risks and limitations	**Unintended behavioral consequences:** incentives for superficial “service behaviors” or scripted interactions aimed at score improvement.
	**Indicator decay:** adaptation to instruments reduces sensitivity over time.
	**Equity/representativeness concerns:** digital feedback may under-represent older, digitally excluded, or socioeconomically disadvantaged patients.
	**Measurability dominance risk:** more substantial reliance on satisfaction scores may overshadow harder-to-measure aspects (clinical appropriateness, long-term outcomes).

#### 3.3.2 Net promoter score (NPS)

NPS is increasingly used as a summary measure of patient loyalty and willingness to recommend a provider or institution. In EDs, NPS provides a rapid, standardized indicator of perceived service quality and responsiveness, complementing traditional satisfaction surveys and enabling near-real-time feedback in fast-paced care environments (119–121).

Table 14 summarizes the technological enablers supporting NPS measurement and discusses their implications.

**Table 14:** NPS: technological enablers and implications for core critiques of KPI use.

Dimension	Content
KPI focus	Loyalty and willingness to recommend; rapid standardized proxy for perceived quality and responsiveness (119–121).
Technological advancements discussed	Digital feedback kiosks and mobile survey platforms (112–114); IoT- and RFID-enabled patient journey tracking linked to experience data (108, 110, 111); sentiment analysis and NLP of qualitative feedback (115–117).
Potential benefits	**Measurability over meaning (partial):** linking NPS to contextual data (waits, crowding) and supplementing with sentiment analysis broadens interpretive depth.
	**Technical and data-related challenges:** digital collection improves timeliness, consistency, and completeness (9, 41).
	**Context and complexity blindness (limited):** integration with journey data supports interpretation relative to system conditions.
Key risks and limitations	**Measurability dominance/oversimplification:** NPS remains reductive; amplification may reinforce reliance on a single-number metric that obscures multidimensional quality, safety, and equity.
	**Unintended behavioral consequences:** surface-level behaviors or selective engagement with likely-positive responders.
	**Indicator decay:** staff learn to influence responses (e.g., prompting after positive encounters).
	**Equity/representativeness concerns:** digital collection may under-represent vulnerable or digitally excluded populations.

#### 3.3.3 Complaint resolution time

Complaint resolution time measures how quickly patient complaints are acknowledged, addressed, and closed, and serves as an indicator of governance quality, transparency, and organizational responsiveness. In EDs, complaint resolution time captures the timeliness of managing concerns arising from urgent or unscheduled care. Efficient complaint handling has been associated with improved trust, reduced litigation risk, and stronger perceptions of accountability (122, 145, 146).

Table 15 summarizes technological approaches to measuring complaint resolution time.

**Table 15:** Complaint resolution time: technological enablers and implications for core critiques of KPI use.

Dimension	Content
KPI focus	Timeliness of complaint acknowledgment and closure; proxy for responsiveness, transparency, and governance quality (122, 145, 146)
Technological advancements discussed	Digital complaint management systems and workflow tracking (122, 145); AI-assisted complaint triage and classification (123–125); RFID/RTLS-enabled staff notification and accountability mechanisms (126, 127); integrated dashboards for governance oversight (9, 80).
Potential benefits (mitigations)	**Technical and data-related challenges:** standardized, timestamped records improve completeness, traceability, and KPI reliability (9, 41).
	**Measurability over meaning (partial):** AI classification and structured workflows capture qualitative complaint dimensions more systematically.
	**Context and complexity blindness (partial):** linking timelines to operational context (staffing, crowding) supports nuanced interpretation.
Key risks and limitations	**Unintended behavioral consequences:** incentives for procedural or superficial closure rather than substantive resolution.
	**Indicator decay:** redefining “resolution” to meet timing requirements erodes validity. **Equity/representativeness concerns:** under-capture from patients with limited digital access or lower health literacy.
	**Measurability dominance risk:** Elevated focus on timing may overshadow deeper learning from narratives essential for improvement.

#### 3.3.4 Sentiment analysis of free-text patient feedback (sentiment-based experience KPI)

Sentiment analysis of free-text patient feedback converts qualitative narratives into indicators of emotional tone, such as satisfaction, frustration, trust, or anxiety. It can provide granular, near–real-time insight into patient perceptions of waiting, communication, and care coordination, particularly in emergency settings (115–117, 128). Enabled by advances in natural language processing and large language models, and supported by digital feedback platforms and integrated dashboards, these approaches extend patient experience measurement beyond traditional surveys and facilitate linkage between experiential data and operational conditions such as congestion and delays (9, 81, 112, 114). By systematically incorporating unstructured narratives, sentiment-based KPIs address aspects of care that are often excluded from conventional metrics and reduce manual analytic burden when combined with scalable digital data capture (25, 41).

Nonetheless, sentiment-based KPIs introduce important limitations. Model opacity poses challenges for interpretability, governance, and accountability, while reducing rich narratives to numeric scores risks reinforcing metric dominance and oversimplified performance judgments (117). These systems may also generate unintended behavioral responses, as staff adapt their communication to influence measured sentiment rather than underlying coordination or safety, and may exacerbate equity concerns by under-representing patients facing literacy, language, or digital access barriers. Over time, such adaptive behaviors can contribute to indicator decay, whereby improvements in sentiment scores do not correspond to substantive gains in care quality or system performance.

### 3.4 Quality of care and patient safety KPIs

Quality of care and patient safety KPIs are used to evaluate clinical effectiveness, safety monitoring, outcome benchmarking, and the identification of preventable harm, thereby supporting evidence-based assessment of whether healthcare delivery achieves intended clinical outcomes without exposing patients to avoidable risk (6–9, 103). These indicators are central to performance measurement frameworks because they link clinical and operational processes to tangible patient outcomes.

Several quality and safety KPIs are widely applied at the hospital level, with closely related equivalents used in ED settings. Each KPI is examined below using a common analytical lens that links technological and data-related advancements to core critiques of KPI-based performance measurement.

#### 3.4.1 Hospital mortality rate

Hospital mortality rate is a core outcome indicator reflecting overall clinical effectiveness and patient safety across inpatient pathways, while its emergency department analogue captures deaths occurring during emergency care and is particularly sensitive to delays in diagnosis, triage, and treatment. Although widely used for outcome benchmarking, mortality indicators are well known to be methodologically fragile due to challenges in case-mix adjustment and contextual heterogeneity (13, 14, 32). Recent technological advancements, including AI-enabled dashboards and predictive risk analytics, continuous vital-sign monitoring through IoT and wearable devices, RFID- and RTLS-based workflow and delay detection, and integrated EHR-based clinical decision support, have enhanced the technical robustness of mortality measurement by improving data completeness, temporal precision, and the real-time availability of mortality-risk signals (9, 52, 66, 81, 84, 86, 95–97, 129, 130). These capabilities strengthen the use of mortality indicators for internal quality improvement by enabling closer linkage between outcomes, physiological deterioration, and upstream workflow conditions, thereby partially addressing context and complexity blindness (9, 41).

Nevertheless, enhanced data capture does not resolve the fundamental limitations of mortality-based performance measurement. Improved instrumentation may increase confidence in inequitable and methodologically fragile benchmarking, as mortality rates remain highly sensitive to case mix, socioeconomic context, referral patterns, and coding practices (13, 14, 32). Moreover, strong emphasis on mortality outcomes risks crowding out other dimensions of performance, including functional outcomes, patient experience, and equity, and may induce unintended behavioral responses such as risk aversion or overly aggressive interventions. Over time, adaptive changes in documentation practices and end-of-life classification can further contribute to indicator decay, eroding the validity of mortality indicators as proxies for sustainable quality and safety performance.

#### 3.4.2 Hospital readmission rate

The hospital readmission rate measures the proportion of patients who experience an unplanned return to the hospital following discharge and is widely used as an indicator of continuity of care, discharge quality, and the adequacy of post-hospital support. Its emergency department analogue, the 72-hour ED return visit rate, captures short-term re-presentations after ED discharge and may signal diagnostic uncertainty, premature discharge, or unresolved clinical issues. Both indicators are highly sensitive to patient complexity and social context and have been shown to generate unintended consequences when embedded in incentive-based performance programs (17, 33, 131–134).

Technological and data-related advancements, including cross-setting EHR integration, AI-enabled readmission risk prediction, telehealth-based follow-up, and IoT-enabled remote patient monitoring, have improved post-discharge visibility and reduced fragmentation across care transitions, supporting more accurate identification and interpretation of readmission and return-visit events (9, 52, 71–73, 95, 130, 135). These tools partially address context and complexity blindness by enabling risk stratification and monitoring relative to patient risk profiles and transition conditions, and by linking readmission events to discharge processes and follow-up pathways (41).

Nevertheless, enhanced technological support does not resolve the core limitations of readmission-based performance measurement. Readmission and ED return rates remain vulnerable to unintended behavioral responses, including avoidance of high-risk patients, premature discharge, or inappropriate diversion strategies. Persistent sensitivity to socioeconomic context and post-acute care availability continues to undermine equitable benchmarking, and improved data capture may inadvertently legitimize unfair comparisons across heterogeneous populations (17, 131). Moreover, strong emphasis on readmission metrics risks crowding out broader outcome domains such as functional recovery and quality of life, while adaptive documentation and classification practices over time may contribute to indicator decay, weakening their validity as proxies for sustainable performance.

#### 3.4.3 Adherence to clinical protocols

Protocol adherence reflects the extent to which evidence-based guidelines are followed during assessment, diagnosis, and treatment, encompassing compliance with standardized care pathways and bundles at the hospital level and time-sensitive protocols, such as sepsis bundles, stroke pathways, and trauma activations, in emergency departments. Variability in adherence has been associated with differences in patient outcomes, highlighting the importance of reliable workflow monitoring and documentation (136, 137). Technological advancements, including EHR-integrated clinical decision support systems, RFID- and RTLS-based workflow monitoring, AI-enabled compliance analytics dashboards, and automated documentation and audit tools, have improved the technical robustness of adherence measurement by enhancing documentation completeness, reducing fragmentation, and enabling more timely and accurate tracking of protocol compliance (9, 66, 81, 86, 90, 91, 94). These systems also partially address context and complexity blindness by allowing deviations from protocols to be interpreted in light of crowding, staffing constraints, and diagnostic delays, and by linking adherence patterns to downstream outcomes and workflow conditions (41).

Despite these improvements, protocol adherence KPIs retain important limitations. Strong emphasis on compliance may promote rigid, checklist-driven behavior that suppresses clinical judgment in complex or atypical cases, while a dominance of measurability can elevate adherence scores over patient preferences or outcome nuance. Over time, adaptive documentation and “box-ticking” practices may preserve measured steps without improving substantive care quality, contributing to indicator decay. Furthermore, structural variation in patient complexity and resource availability across settings continues to limit equitable benchmarking, as technologically enhanced adherence metrics may legitimize fragile comparisons rather than reflect genuine differences in performance.

#### 3.4.4 Complication-related indicators

Complication-related indicators capture adverse events such as hospital-acquired infections, procedural complications, and medication-related harm, with emergency department analogues reflecting adverse events occurring during or shortly after emergency care, including procedural errors or delay-related harms. These indicators are central to identifying preventable harm and informing quality improvement efforts, while also highlighting persistent challenges in attributing complications to specific settings, processes, or phases of care (25, 103). Recent technological developments, including EHR-integrated adverse event detection and surveillance systems, AI-enabled safety analytics and anomaly detection, IoT- and sensor-based infection control and environmental monitoring, and RFID- and RTLS-based workflow and process tracing, have enhanced the technical fidelity of complication measurement by improving detection, reducing underreporting, and strengthening documentation consistency relative to voluntary reporting mechanisms (9, 52, 66, 81, 86, 94, 105, 107, 138). By linking adverse events to workflow patterns, environmental conditions, and operational stressors, these tools partially address context and complexity blindness and expand the conceptual scope of harm indicators to include latent and indirect risks (25, 41).

Despite the advances, fundamental limitations persist. Increased detection without corresponding improvements in attribution can intensify interpretive ambiguity and complicate accountability, while rising recorded complication rates may be misinterpreted as deteriorating performance or provoke defensive responses (103). Surveillance-driven measurement may also encourage risk-averse behavior or defensive documentation practices. Over time, organizations may optimize reporting workflows to reduce recorded complications rather than underlying risk, contributing to indicator decay. Moreover, complication-related indicators remain sensitive to patient complexity, comorbidities, and contextual factors, limiting equitable benchmarking across heterogeneous settings. More broadly, across quality and patient safety KPIs, technological enhancements improve measurement completeness, timeliness, and process linkage but do not resolve enduring challenges of risk adjustment, attribution, and contextual heterogeneity and may amplify metric dominance and unintended consequences when KPIs are embedded in incentive structures rather than governed as learning tools (9, 13, 14, 32, 41, 103).

### 3.5 Workforce-related KPIs

Workforce-related KPIs are used to assess staffing adequacy, workload balance, and workforce performance, supporting workforce sustainability, burnout risk mitigation, operational resilience, and continuity of care across hospital services (9, 80, 139). These indicators are critical because workforce capacity and deployment strongly influence care quality, patient safety, and system responsiveness, particularly in high-pressure clinical environments.

Several workforce KPIs are commonly applied at the hospital level, with closely related equivalents used in ED settings. Each KPI is examined below through a standard analytical lens that links technological and data-related advancements to core critiques of KPI-based performance measurement.

#### 3.5.1 Staff-to-patient ratio

The staff-to-patient ratio is a foundational indicator of staffing adequacy and workload distribution across inpatient wards and clinical units. Its ED analogue reflects alignment between patient demand and available staff in fast-paced, unpredictable environments. Inadequate ratios are associated with care delays, reduced throughput, and a higher risk of adverse events, whereas balanced staffing supports patient outcomes and staff well-being (139, 140). Technological developments, including RTLS- and RFID-based workforce tracking, AI-enabled workforce analytics and demand forecasting, and integrated dashboards linking staffing, patient flow, and outcomes, have improved the operational relevance of this indicator by providing real-time visibility into staff presence and patient load, thereby reducing reliance on static rosters (9, 66, 80, 81, 87). These tools partially address context and complexity blindness by enabling staffing ratios to be interpreted relative to acuity, demand surges, and spatial constraints, and by linking nominal ratios to functional performance and outcomes (41).

Despite improved measurement fidelity, staff-to-patient ratios remain an imperfect proxy for workforce adequacy. Threshold-driven redeployments aimed at meeting ratio targets may overlook workload intensity, skill mix, and staff fatigue, while a strong emphasis on ratios risks crowding out critical workforce dimensions, such as burnout, morale, and cognitive load. Over time, adaptive changes in role definitions or counting practices can inflate reported ratios without increasing effective capacity, contributing to indicator decay. Moreover, persistent sensitivity to unit type, patient complexity, and care context limits equitable benchmarking across settings, as technologically enhanced ratios may continue to support methodologically fragile comparisons.

#### 3.5.2 Workforce availability

Workforce availability refers to the real-time presence and accessibility of clinical staff. It is increasingly monitored using digital tracking technologies to assess coverage, identify bottlenecks, and support dynamic responses to demand surges. In high-variability settings characterized by frequent interruptions and fluctuating workloads, such visibility is intended to enhance coordination and operational resilience (66, 87, 147). Technological approaches, including RTLS- and RFID-based staff tracking, integrated workforce management and operational dashboards, and AI-enabled demand sensing and surge prediction, have improved the technical fidelity of availability measurement by replacing static schedules with real-time presence data, thereby increasing accuracy, timeliness, and operational relevance (9, 41, 80, 81, 139). These systems partially address context and complexity blindness by enabling availability to be interpreted relative to spatial dispersion, interruptions, and fluctuating demand. They can support limited forms of operational resilience by facilitating rapid responses to surges and disruptions.

However, workforce availability metrics introduce important risks. Perceived surveillance associated with continuous tracking may undermine trust, autonomy, and morale, particularly in high-pressure clinical environments. Emphasis on visible presence risks overshadowing less observable but critical dimensions of work, such as cognitive load, skill mix, and task complexity. Over time, staff may adapt their behavior to “appear available,” for example, by remaining within monitored zones without improving responsiveness, thereby contributing to indicator decay. Moreover, persistent contextual differences, such as higher interruption rates, teaching responsibilities, or greater patient complexity, limit equitable benchmarking across units, as technologically enhanced availability indicators may continue to support methodologically fragile comparisons.

#### 3.5.3 Training and protocol compliance

Training and protocol compliance reflects the extent to which staff complete required training and adhere to evidence-based protocols, with particular importance in emergency care settings where time-critical pathways such as sepsis, trauma, and stroke have immediate safety implications. Variability in training uptake and protocol adherence has been linked to inconsistent care delivery, underscoring workforce development as a core component of quality and safety management (136, 137). Digital technologies, including EHR-integrated decision support and protocol checklists, online training platforms and learning management systems, RFID- and RTLS-based workflow monitoring linked to training outcomes, and AI-enabled analytics for detecting compliance patterns, have improved the technical robustness of measurement by reducing reliance on self-reporting and retrospective audits and by enhancing completeness, accuracy, and timeliness of compliance data (9, 41, 66, 81, 86, 90, 94, 141). By linking training completion to real-world protocol execution, these tools partially shift measurement from nominal participation toward applied competence and allow deviations to be interpreted relative to operational pressures such as crowding, staffing shortages, and interruptions.

Nevertheless, technology-enabled compliance metrics retain important limitations. Emphasis on formal completion may encourage “check-the-box” training or rigid protocol adherence that displaces clinical judgment in complex cases, while a metric-driven approach risks overshadowing teamwork, experiential learning, and adaptive expertise. Over time, superficial engagement with training modules and adaptive documentation practices can contribute to indicator decay, weakening their validity as measures of workforce capability. Moreover, persistent contextual differences in workload intensity and access to training opportunities limit equitable benchmarking, as technologically enhanced compliance indicators may legitimize fragile comparisons across units and hospitals operating under heterogeneous conditions. More broadly, across workforce KPIs, technological advancements primarily mitigate technical data limitations but may also amplify surveillance effects, threshold-driven behavior, and metric dominance if not embedded within governance frameworks that prioritize human-centered implementation and learning over control (9, 33, 41).

### 3.6 Resource utilization and operational efficiency KPIs

Resource utilization and operational efficiency KPIs are used to assess how effectively physical, technological, and environmental resources are deployed, supporting capacity optimization, waste reduction, infrastructure efficiency, and environmental safety across hospital systems (9, 68, 103). These indicators are central to operational performance management because inefficient resource use directly contributes to congestion, higher costs, and compromised care delivery.

Several resource utilization KPIs are widely applied at the hospital level, with closely related equivalents used in ED settings. Each KPI is examined below through a common analytical lens that links technological and data-related advancements to core critiques of KPI-based performance measurement.

#### 3.6.1 Bed occupancy rate

The bed occupancy rate measures the intensity of inpatient bed utilization and is widely used as a proxy for capacity management, flow balance, and coordination between admission and discharge processes. In emergency departments, related constructs such as crowding and treatment-space occupancy capture the availability of care spaces and strongly influence waiting times, throughput, and patient safety. Persistently high occupancy levels in either setting are associated with congestion, delayed care, and increased risk of adverse events (98, 99). Technological advancements, including RFID- and RTLS-based bed and patient flow tracking, integrated EHR-based bed management systems, AI-enabled capacity prediction and surge management tools, and IoT-enabled environmental and space-utilization monitoring, have improved the technical accuracy and timeliness of occupancy measurement by reducing reliance on manual census systems and delayed reporting (9, 41, 66, 81, 86, 87, 90, 91, 105, 107). These systems partially address context and complexity blindness by enabling occupancy levels to be interpreted relative to discharge delays, diagnostic bottlenecks, staffing constraints, and demand surges, and by strengthening coordination between hospital wards and emergency departments to reduce boarding and congestion.

Despite improved measurement fidelity, bed occupancy indicators retain important limitations. Efficiency-oriented optimization may prioritize high utilization while neglecting patient acuity, clinical appropriateness, and the need for buffer capacity to maintain safety. Technology-enabled occupancy management can also generate unintended behavioral responses, including premature discharge, inappropriate transfers, or congestion shifting through boarding practices. Over time, adaptive redefinitions of what constitutes an “occupied” or “available” bed may improve reported performance without improving underlying flow or safety, contributing to indicator decay. Moreover, persistent sensitivity to case mix, referral patterns, and post-acute care availability limits equitable benchmarking, as technologically enhanced occupancy metrics may continue to support methodologically fragile comparisons across heterogeneous settings.

#### 3.6.2 Equipment utilization

Equipment utilization measures the extent to which medical devices and diagnostic equipment are actively used relative to their availability and is particularly salient in emergency departments where time-critical assets such as imaging devices, monitors, and infusion pumps directly influence diagnostic and treatment timeliness. Limited visibility or inefficient use of such equipment can create workflow bottlenecks, delay care, and drive unnecessary capital expenditure, whereas improved utilization supports faster service delivery and cost containment (64–66). Digital technologies, including RFID- and RTLS-based equipment tracking, IoT-enabled device usage monitoring and analytics, and integrated asset management dashboards, have strengthened the technical robustness of utilization measurement by providing objective, real-time data and reducing reliance on inaccurate inventory records (9, 41). These tools partially address context and complexity blindness by allowing utilization to be interpreted relative to clinical demand and patient flow, and by distinguishing workflow-induced idle time from genuine overcapacity, thereby adding explanatory depth to efficiency assessments.

Nevertheless, equipment utilization KPIs retain essential limitations. Strong emphasis on maximizing device use may incentivize unnecessary testing or overuse to justify capital assets, while operational pressures can promote local hoarding or selective deployment that prioritizes utilization over clinical appropriateness. Over time, adaptive changes in activation or recording practices may inflate reported utilization without improving timeliness or outcomes, contributing to indicator decay. Moreover, utilization metrics remain sensitive to institutional role and configuration, for example, trauma centers vs. community hospitals, limiting equitable benchmarking across heterogeneous settings, even when technologically enhanced.

#### 3.6.3 Diagnostic turnaround time

Diagnostic turnaround time captures the interval required to complete and report laboratory tests or imaging studies and is widely used as an indicator of diagnostic efficiency and care coordination. In emergency departments, timely diagnostics are particularly critical, as delays prolong length of stay, impede clinical decision-making, and contribute to crowding and reduced throughput (99, 101). Technological developments, including IoT-enabled laboratory and imaging workflow tracking, RFID- and RTLS-based monitoring of samples and patient movement, AI-enabled diagnostic prioritization and workload balancing, and integrated EHR–laboratory–radiology information systems, have improved the technical robustness of turnaround-time measurement by reducing fragmentation, enabling real-time tracking, and limiting reliance on retrospective reporting (9, 41, 66, 81, 86, 90, 100). These systems partially address context and complexity blindness by allowing delays to be interpreted relative to competing demand, staffing constraints, and equipment availability, and by supporting a more predictable turnaround that can reduce length of stay, crowding, and downstream congestion.

Despite these advances, diagnostic turnaround time remains an imperfect proxy for performance. Strong emphasis on speed may compromise diagnostic appropriateness and quality. It can encourage unnecessary testing or rushed interpretation, while operational targets may incentivize prioritizing simpler tests over more complex or clinically meaningful diagnostics. Over time, adaptive redefinitions of completion or reporting points may preserve apparent performance without improving underlying efficiency, contributing to indicator decay. Moreover, persistent sensitivity to case mix, test complexity, and institutional resource availability limits equitable benchmarking across settings, even when measurement is technologically enhanced.

#### 3.6.4 Asset downtime

Asset downtime measures the proportion of time medical equipment is unavailable due to maintenance, malfunction, or misplacement and has particular operational significance in emergency departments, where unavailable assets can delay assessment, diagnostics, or treatment in time-sensitive cases. Monitoring downtime supports preventive maintenance strategies and contributes to operational resilience in high-demand environments (66, 80). Digital approaches, including RFID- and RTLS-based asset tracking, IoT-enabled predictive maintenance and condition monitoring, and integrated asset management and maintenance dashboards, have strengthened the technical fidelity of downtime measurement by reducing reliance on manual logs and reactive reporting and by improving the completeness, accuracy, and timeliness of availability data (9, 41, 66, 80, 142). These tools partially address context and complexity blindness by allowing downtime to be interpreted relative to usage intensity, maintenance cycles, and demand surges, and by supporting faster recovery from unplanned failures in emergency settings.

Nonetheless, asset downtime indicators retain essential limitations. Strong emphasis on minimizing downtime may overlook clinical criticality, redundancy requirements, or safety-mandated maintenance intervals. It may incentivize unintended behaviors such as deferring maintenance, applying superficial fixes, or continuing to use marginal equipment to preserve availability statistics. Over time, adaptive reclassification and reporting practices can improve apparent performance without enhancing reliability, contributing to indicator decay. Moreover, persistent sensitivity to equipment age, capital replacement cycles, and institutional role limits equitable benchmarking across organizations, as technologically enhanced downtime metrics may continue to support methodologically fragile comparisons.

#### 3.6.5 Indoor air quality (IAQ) indicators

IAQ indicators are increasingly recognized as measures of environmental safety, infection control, and staff and patient well-being. In emergency departments, IAQ is particularly salient due to high patient occupancy, rapid patient turnover, and frequent use of aerosol-generating procedures, with poor air quality associated with elevated infection risk and reduced comfort (138, 143). Technological advancements, including IoT-enabled environmental sensing, IAQ dashboards integrated with building management systems, and AI-enabled anomaly detection, have expanded the scope and technical fidelity of IAQ measurement by enabling continuous, high-frequency monitoring and real-time linkage between environmental conditions, occupancy, and ventilation performance (9, 41, 81, 105, 107, 138). By incorporating environmental determinants into performance assessment, IAQ indicators substantively address critiques that traditional KPI frameworks neglect systemic and contextual factors influencing safety and well-being (25, 103).

Nevertheless, IAQ-based KPIs present important limitations. Simplified composite scores may obscure spatial variability and short-term exposure risks, while compliance-driven responses can encourage localized or superficial adjustments that fail to address structural drivers such as chronic overcrowding or aging infrastructure. Over time, optimization toward predefined thresholds rather than actual risk reduction may undermine the informational value of IAQ indicators, contributing to indicator decay. Moreover, persistent sensitivity to building age, design, and investment levels constrains equitable benchmarking across organizations, as technologically enhanced IAQ metrics may continue to support methodologically fragile comparisons. More broadly, across resource utilization and efficiency KPIs, technological advancements primarily mitigate technical and visibility constraints but do not resolve underlying structural and contextual determinants of performance and may amplify metric dominance, gaming behaviors, and indicator decay when efficiency targets are pursued without safeguards for clinical appropriateness, safety, buffer capacity, and equity (9, 25, 41, 68, 103).

### 3.7 Discussion and implications

The findings of this review both align with and extend prior research on KPI-based performance measurement and digital health transformation. Consistent with established critiques of performance regimes, the analysis confirms that KPIs remain vulnerable to gaming, inequitable benchmarking, and indicator decay (11, 19, 28). Likewise, concerns regarding the methodological fragility of outcome indicators, such as mortality and readmission rates, persist despite improvements in data availability and analytic sophistication (13, 14).

However, in contrast to strands of digital health literature that emphasize enhanced data quality, real-time monitoring, and predictive analytics as direct drivers of performance improvement (45, 46), this synthesis demonstrates that technological advancements primarily address technical and informational constraints rather than the structural limitations of KPI regimes. Improved instrumentation strengthens measurement precision, but it does not fundamentally resolve incentive distortions, contextual heterogeneity, or governance fragilities. By conceptually linking established critiques of KPI regimes with technological infrastructures across performance domains, this review advances a governance-centered perspective on digital performance measurement.

#### 3.7.1 Theoretical implications

From a theoretical standpoint, this study contributes by distinguishing between technical measurement constraints and structural governance distortions in KPI-based performance regimes. While much of the digital health literature implicitly assumes that better data leads to better performance, the present synthesis demonstrates that improvements in interoperability, analytics, and real-time dashboards do not automatically translate into improved organizational behavior or equitable benchmarking. This reframing positions technology not as a corrective mechanism for KPI limitations, but as a structural amplifier whose effects depend on incentive design and governance architecture. In doing so, the study extends existing critiques of performance regimes (19, 28) into the era of digital transformation and contributes to broader debates on algorithmic governance and data-driven management.

#### 3.7.2 Practical implications

The findings also carry significant implications for practitioners. First, digital investments should not be conflated with performance reform. While technology enhances measurement fidelity and operational visibility, it does not redefine value priorities or eliminate unintended incentive effects. Hospital executives and policymakers should therefore approach AI-enabled dashboards, interoperability systems, and predictive analytics tools as instruments embedded within governance regimes, rather than as stand-alone solutions to performance challenges.

Second, governance design and incentive alignment remain decisive in determining whether technologically enhanced KPIs function as tools for organizational learning or mechanisms of control. Without adaptive review mechanisms, context-sensitive benchmarking, and safeguards against metric dominance, increased data granularity may intensify gaming behaviors, inequitable comparisons, and indicator decay.

Third, performance oversight bodies and digital transformation teams should implement structured KPI review cycles and incorporate qualitative and contextual indicators alongside quantitative dashboards to prevent over-reliance on narrow efficiency metrics. In high-stakes domains such as mortality, readmissions, and throughput management, particular attention should be given to case-mix sensitivity and socioeconomic context to avoid reinforcing structural inequities (13, 14).

Overall, the analysis suggests that technology acts less as a transformer than as an amplifier of KPI systems: it increases visibility and responsiveness, yet its ultimate impact depends on how performance regimes are structured, interpreted, and governed.

This conclusion is consistent with longstanding critiques of KPI-based performance systems that emphasize gaming dynamics, inequitable benchmarking, and performance paradox effects (19, 28, 30). These studies similarly demonstrate that measurement refinement alone does not resolve structural distortions embedded in incentive regimes.

At the same time, the present synthesis contrasts with more technology-optimistic perspectives that portray digital health infrastructures, interoperability systems, and AI-enabled analytics as corrective solutions to performance measurement challenges (45, 46). While such advancements clearly improve data fidelity and operational transparency, the findings here suggest that they primarily address technical constraints rather than the governance structures that shape the effects of KPIs.

## 4 Conclusions and future research

This review examined whether technological and data-related advancements can improve the use of KPIs in hospital management by analyzing six recurring critiques of KPI-based performance measurement across multiple performance domains. The findings suggest that while a common overarching conclusion emerges, its implications vary across critiques and KPI categories.

### 4.1 Answers to the research questions

To synthesize the analysis and clarify the implications, the two guiding review questions are addressed explicitly below.

First, are technical and data-related challenges a key barrier to effective KPI use?

The findings indicate that they are. Across the financial, timeliness and access, patient experience, quality and safety, workforce, and resource utilization domains, fragmented information systems, incomplete data capture, limited interoperability, and insufficient contextual variables undermine the reliability and interpretability of indicators (9, 41). Technological advancements consistently mitigate these technical constraints by improving data quality, granularity, timeliness, and integration. In this respect, technical limitations represent a genuine obstacle to the effective implementation of KPI systems.

Second, can technological advancements resolve the core limitations of KPI-based performance regimes?

The review demonstrates that the answer is more nuanced. While technology enhances measurement robustness, it does not eliminate structural distortions embedded in incentive design, benchmarking practices, and governance architectures (144). For critiques related to unintended behavioral consequences, inequitable benchmarking, context and complexity blindness, and indicator decay, technological enhancement produces heterogeneous effects. In several domains, particularly financial and timeliness indicators, real-time analytics and automated dashboards may intensify metric salience and threshold-driven behavior, reinforcing gaming and tunnel vision rather than supporting organizational learning (11, 19). By contrast, system-aware analytics and simulation-based approaches show greater potential to address contextual complexity, but only when employed as exploratory decision-support tools rather than simplified performance targets (33, 34).

A central conclusion of this review is that technology does not create value as a standalone intervention (50). While digital infrastructures strengthen measurement systems, they do not define what constitutes meaningful performance. When KPIs are weakly aligned with clinical, organizational, or societal value, technological enhancement risks optimizing metrics rather than improving outcomes, an issue increasingly emphasized in performance management and health policy research (25, 28). This misalignment helps explain why improved measurement frequently fails to translate into sustained performance improvement and may even accelerate indicator decay.

From a practical perspective, these findings imply that investments in analytics and digital health technologies should be guided by value-oriented performance objectives rather than measurement improvement alone. KPIs should be governed as diagnostic instruments that support contextual interpretation, trade-off analysis, and organizational learning, rather than as rigid targets of control. Adaptive KPI governance, including regular indicator review, triangulation with qualitative insights, and explicit recognition of contextual constraints, is essential to prevent metric dominance and unintended behavioral responses (11, 41).

### 4.2 Limitations and future research

This review is subject to limitations inherent in its critical-conceptual design, which prioritizes analytical integration over exhaustive empirical coverage (54, 57). First, the thematic mapping of KPI critiques and technological enablers involves interpretive judgment, and alternative classifications may be possible. As with all critical–conceptual syntheses, this approach may be perceived as subjective because it relies on interpretation rather than formal statistical aggregation. Second, the review does not follow a fully systematic protocol; while the search process is transparent, the synthesis is illustrative rather than comprehensive (50). Third, the analysis is conceptual and does not empirically test the proposed “technology as amplifier” argument. Future empirical and longitudinal studies are needed to examine how digitally enhanced KPI systems evolve under different governance and incentive regimes. Finally, given variations in healthcare systems and the rapid evolution of digital technologies, the findings should be interpreted with contextual caution.
